# The politicizing clinic: insights on ‘the social’ for mental health policy and practice

**DOI:** 10.1007/s00127-023-02573-2

**Published:** 2023-12-18

**Authors:** Dominique P. Béhague, Helen Gonçalves, Suélen Henriques da Cruz, Larissa de Cruz, Bernardo L. Horta, Natália P. Lima

**Affiliations:** 1 Vanderbilt University, Medicine Health, and Society, Nashville, Tennessee USA; 2 Postgraduate Program in Epidemiology, Federal University of Pelotas, Pelotas, Brazil; 3 Kings College London, Social Medicine & Global Health, London, UK

**Keywords:** Adolescence, Mental health care, Social determinants, Political therapeutics, Psychiatric reform

## Abstract

**Purpose:**

In this paper, we explore how Brazilian socially sensitive therapy can respond to care-users’ desire to change the social and political forces shaping their lives. We use this case to demonstrate the limits of the “social determinants of health” agenda which, when operationalized, tends to leave questions of lasting structural change aside.

**Methods:**

We report on mixed methods ethnographic and epidemiological results from the 1982 Pelotas (Brazil) birth cohort study, a prospective study of 5914 children. Ethnographic analysis explored the cyclical relationship between schooling, mental health care, conceptualizations of mental distress, social and political engagement, and experiences with diverse forms of discrimination. Epidemiological bivariate and multivariate analyses examined differences in socio-political participation and the reporting of discrimination at different time-points for participants who used therapy with those who did not. Effect modification analysis tested the hypothesis that the socially empowering effects of therapy were greater for marginalized and minoritized youth.

**Results:**

Most young people living in situations of precarity experienced therapy, particularly when based in schools, to be a blame-inducing process. A more fulfilling and impactful therapeutic experience took shape when young people were able to shift the focus away from symptom reduction and behavioral management toward narrative life analyses, social debate, and political agency. Use of socially sensitive therapy was statistically associated with increased political participation and reporting of discrimination after controlling for confounders. The empowering effects of therapy were greater for those with less formal education and family income, but not for young people who identified as black, brown, or non-white.

**Conclusion:**

The findings underscore the importance of considering agency, sociality, and politics when theorizing “the social” in clinical practice, and health and social policy.

## Introduction

The “social determinants of health” framework has gained considerable traction over the past decades [1]. Epidemiologists have documented the role that social “risk factors” play in increasing the likelihood of mental illness [2, 3]. In global health, attention to the social realm has contributed to reducing inequities in access to mental health care [4]. Efforts have been made to train providers to be sensitive to the economic, gendered, and racialized conditions impacting patients’ lives [5], and the growing prominence of anthropological research in public health has brought attention to the limits of behaviorist and pharmacological treatments in mental health, as well as to people’s everyday experiences of structural inequities [6].

While interest in the social has become more mainstream, several challenges remain. Social determinants are typically measured and operationalized through individual-level demographic characteristics and behaviors [7–10]. This tends to lead to the prioritization of so-called downstream interventions, such as targeted biological or health education interventions and reduction of inequities in access to services [11]. While useful, these approaches neglect the structures that produce health inequities in the first place. In the absence of sustainable enabling environments, downstream interventions can put undue pressure on or even blame individuals for systemic failings [12–15]. Furthermore, in qualitative health research, attention to the social often translates into emphasizing first person narratives of suffering, which can paint an intractable view of the social and a pacifying view of individuals [16–18].

Some answers to these limitations can be found in Latin American social medicine (LASM), known as “collective health” in Brazil, which first took shape in the 1970s in academia, public health, and grass roots organizations. Rather than focus on “social determinants,” LASM scholars shed light on the “processes of determination” that produce health and illness, such as wealth accumulation, reproduction of social hierarchies, and the political systems that justify late capitalism [19, 20]. The emphasis on *processes* rather than *determinants* or *structures* eschews a reductionist, individualized, and depoliticized view of the social while also frontloading questions of change [21–23]. In theorizing change, LASM scholarship avoids bifurcating “upstream” policy-making from “downstream” intervention, focusing instead on the synergies between activism, institutional reform, governmental policy-making, and everyday social relations [24].

Building on these insights, this paper explores the change-making potential of socially sensitive psychotherapy using a conceptualization of the social that emphasizes agency, sociality, and politics [13, 25]. Drawing from the 1982 Pelotas Birth Cohort, an interdisciplinary longitudinal study directed by researchers at the Federal University of Pelotas in Southern Brazil [26–28], our aim is to demonstrate how transformative processes can unfold as patients, or users of care, take the lead in their therapeutic journey and seek to change the forces shaping their lives. We consider how mental health care can and should draw from social justice principles and practice. Community-based providers have made a compelling case for their role as agents of social—and not just individual—change [29, 30]. For many, this includes challenging the role that psychiatric institutions have played in perpetrating harm and reproducing inequities [31].

Brazil provides an opportune context in which to explore the relationship between psychotherapy and social change. Following the end of the military dictatorship in 1984, the decades of the 1990s and 2000s—precisely when the 1982 cohort participants were coming of age—witnessed large-scale economic, welfare, and health reforms [32]. In education, new policies widened access to public schooling and school-based counselling. In health, psychiatric hospitalization rates were reduced to curb harmful forms of institutionalization and make way for state-funded community-based mental health care [33]. Those leading reform, inspired by long-standing schools of psychoanalysis, social medicine, liberatory pedagogy, and anti-psychiatry writers such as Italian Franco Basaglia [31, 34], were explicitly critical of the reductionist behavior-based orientations and pharmaceutical treatments that were at the time increasing in countries such as the USA and the UK.

## Methodology

**Fig. 1:**
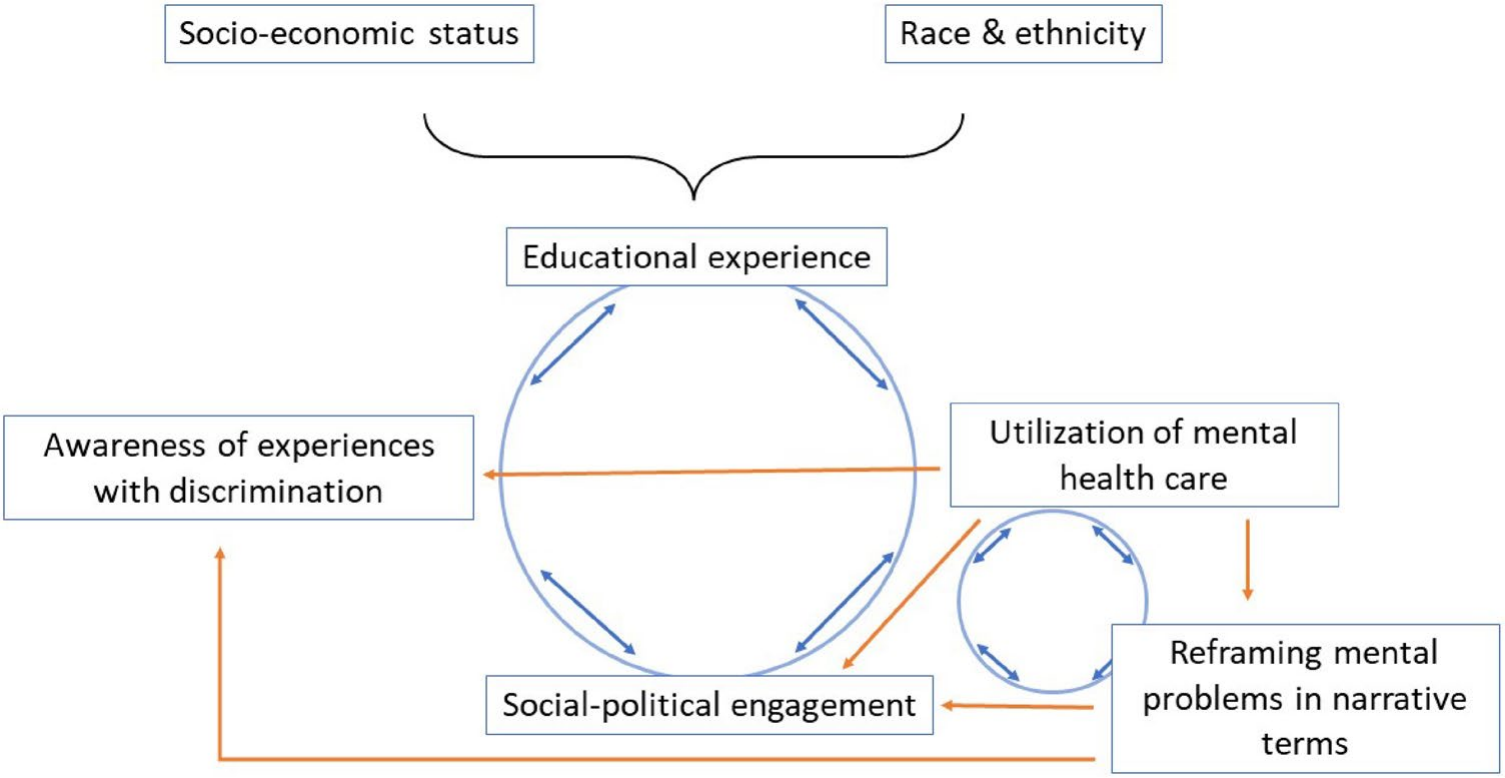
Conceptual model

Bringing ethnographic and epidemiological data from the cohort together, our methodology proceeded through iterations of inductive and deductive methods. Inductive methods were used to remain open to unexpected findings, identify new concepts, variables, and hypotheses, and provide interpretive depth. Deductive methods were used to test new hypotheses and address generalizability of ethnographic findings [35]. Results from this process are summarized in the figure, which served as a guide for the analyses (Fig. 1).

The ethnography centered on the cyclical relationship between schooling, mental health care, conceptualizations of mental distress, socio-political engagement, and experiences with diverse forms of discrimination, depicted in blue in the figure. Ethnographic results gave rise to two epidemiological hypotheses, depicted in orange: (1) use of mental health care is associated with increased reporting of discrimination and political participation, and these associations remain significant after accounting for key measurable confounders. (2) Young people who sought to reframe their problems in psychodynamic and narrative terms—and were allowed or encouraged to do so—experienced a socially and politically empowering form of therapy more frequently than those who did not go to therapy, and than those who understood their problems in behavioral terms. Given our interest in the potential of the clinic, the epidemiological analysis focuses on only one direction of these associations, though ethnographic results suggest these are likely bidirectional. Comparative ethnographic analysis gave rise to a third hypothesis explored with effect modification: (3) the socially and politically empowering effects of therapy were greater for marginalized and minoritized youth.

### Methods

Representative subsamples of the epidemiological cohort (*N* = 5914 at birth) have been followed up at regular intervals, with the most recent follow-up in 2012–13 [26, 36]. Epidemiological variables used in this paper were collected in: the 1982 base-line survey; the 2001 follow-up survey conducted with a sample living in a random selection of 27% of the census tracts in the city; the 2004–05 follow-up, which sought to interview all individuals in the original cohort; the 2006 follow-up, which took a random sample of 25% of the original cohort; and the 2012–13 follow-up, which aimed to interview the whole cohort. Loss to follow-up for the 2001, 2004–05, 2006, and 2012–13 surveys was 21%, 23%, and 32%, respectively [26].

The ethnographic component of the cohort was initiated in 1997 when participants turned 15. A sub-sample of 96 mother–child pairs was selected at random from the larger birth cohort study and visited intermittently, with intensive periods of fieldwork in 1997–1999, 2000–01, 2005–06, 2007, and again starting in 2020, by authors DB, HG, and SC, and a team of 4 research assistants [37]. Methods included participant observation and repeated semi-structured and informal interviewing with youth, their mothers, and other family members and friends. The lead author also conducted semi-structured interviews with over 100 local experts, including mental health care providers, public health officials, and policymakers. All interviews were tape-recorded, transcribed, and anonymized.

At each follow-up, ethics approval was obtained from the Federal University of Pelotas’ Faculty of Medicine (UFPel) ethics board; informed consent was elicited from participants at each of these. When cohort children were under 18 years of age, informed consent was obtained from parents and children; once over 18 years of age, informed consent was obtained only from cohort participants. The ethnographic follow-up starting in 2020 was also approved by Vanderbilt University (IRB #191881).

### Epidemiological measures

#### Exposures

Measures of use of psychotherapy were collected in 2001, 2004–05, 2006 and 2012–13. At each follow-up, participants were asked if they had visited a psychologist or psychiatrist in the last year, and in 2001, they were also asked about lifetime use. Given our interest in how mental distress is framed, the 2001 survey used an open-ended question to ask young people to name the problem for which they needed therapy. Building on insights from the ethnographic study, answers were grouped into two main categories: (1) *behavioral*: learning difficulties, school problems, inattention, hyperactivity, aggression, conduct problems, externalizing behaviors, and (2) *narrative*: difficult life events, damaging constructions of the self, and nerves, stressful, and depressive episodes links to specific life conditions. Significant intellectual disability and Down syndrome were excluded. We take this variable to indicate long-standing differences in how clinicians and researchers frame mental distress, in either cognitive behavioral ways or through open-ended psychodynamic and narrative methods [38].

#### Outcomes

As will be elaborated on in the results section, participants in the ethnography, especially those who challenged behavioral framings, articulated a view of the social that emphasized relational dynamics, such as conflict and discrimination, more so than poverty or characteristics relating to identity [39, 40]. Thus, in the 2006 and 2012–13 surveys, we asked participants if they had experienced discrimination in relation to race, income, religion, sexual orientation, gender and/or disability in the year prior to the survey interview. Answers to these yes/no questions were grouped into a new dichotomous variable: none or one or more forms of discrimination. Research suggests that oppressed individuals often experience discrimination without identifying it as such [41]; thus, we consider this variable to also be a proxy for the capacity or willingness to name discrimination as such.

Given our interest in the empowering potential of therapy, we measured socio-political activity in three of the surveys as shown in Table 1.

**Table 1:** Measures of political activity

2001	2004–05	2006
Voted at 16 or 17 years of age^a^ Ever participated in political protest or signed a petition	Ever participated in neighborhood association Ever participated in union/employer’s syndicate Ever participated in protest or petition Ever participated in political campaign Ever participated in political groups at school/university or relating to political party Ever participated in youth groups	Ever participated in political protest or signed a petition

^a^Voting between 16 and 18 years of age is optional and obligatory starting at age 18

### Analysis

Following an abductive theory-generating approach, the ethnographic material was coded according to pre-existing and emerging themes, giving particular attention to dynamic theories of the social [42]. Summary tables of aggregate results were used to identify patterns. For the epidemiological hypotheses, bivariate and multivariate regression were used. In the regression analysis, confounders included: family income at birth, maternal education at birth, household asset index in 1984, participant’s self-identified race and family income at most recent follow-up. We used Poisson regression with robust adjustment of the variance to estimate the relative risk. Effect modification analysis was conducted by estimating separate RRs for each modifier, and then, carrying out Chi-squared tests of interaction to check for heterogeneity of these RRs; the degrees of freedom for this equal the number of levels minus one. All analyses were carried out using SPSS v 28.

## Results

A comparison of the ethnographic sample with the larger cohort at each follow-up and according to key demographic variables—gender, race, income, education—shows a similar degree of heterogeneity in both ethnographic and epidemiological samples (data not shown). Frequencies of main exposures and outcomes variables for the epidemiological and ethnographic samples can be found in Table 2.

**Table 2:** Prevalence of main exposures and outcomes

Variable	Epidemiological sample		Ethnographic sample	
	*n*	%	*n*	%
Main exposures				
Lifetime use of therapy (2001)				
None	903	70	62	67
Some	382	30	30	33
Framing used in explaining need for therapy at any point up to 19 years (2001)				
None	903	73	62	69
Behavioral	152	12	12	13
Narrative	186	15	16	18
Use of therapy in last year (2001)				
No	1189	93	86	90
Yes	92	07	06	06
Use of therapy in last year (2004–05)				
No	1083	93	73	92
Yes	80	07	06	08
Use of therapy in last year (2012–13)				
No	857	88	67	93
Yes	116	12	05	07
Main outcomes				
Relays having experienced one or more forms of discrimination (2004–05)				
No	1024	88	73	87
Yes	136	12	11	13
Relays having experienced one or more forms of discrimination (2012–13)				
No	797	82	59	82
Yes	178	18	13	18
Voted at 16 or 17 years of age? (2001)				
No	764	54	49	49
Yes	656	46	49	49
Ever participated in political protest or signed a petition (2001)				
No	788	60	63	63
Yes	465	35	35	35
Ever participated in neighborhood association (2004–05)				
No	1125	97	76	96
Yes	36	3	03	04
Ever participated in union/employer’s syndicate (2004–05)				
No	1127	97	75	95
Yes	34	23	04	05
Ever participated in protest or petition (2004–05)				
No	790	68	54	68
Yes	371	32	25	32
Ever participated in formal political campaign (2004–05)				
No	963	83	66	85
Yes	198	17	13	17
Ever participated in political groups at school/university or political party (2004–05)				
No	1001	86	65	82
Yes	160	14	14	18
Ever participated in youth groups (2004–05)				
No	814	70	55	70
Yes	347	30	24	30
Ever participated in political protest or signed a petition (2006)				
No	509	53	47	51
Yes	461	48	45	49

In the 2001 survey, 30% of all participants reported having seen a psychologist, psychiatrist, or social worker at some point in their life. Utilization rates ranged from 26% for those with the lowest household income (US$ 100 to 300 dollars per month) to 41% for those with the highest income ($600 + dollars per month), using family income measured in 1982. About 35% of these services were provided in the private sector and 65% in the public sector, in either clinics or schools. These data show that health care reform achieved a high degree of access to public services.

### The chronicity of conflict

In interviews, clinicians explained that ideally, psychodynamic principles should be adhered to, even in the more resource constrained context of the public sector. This meant refraining from categorically diagnosing care-users and instead, using free association techniques to analyze the meaning and “function” of their symptoms. Echoing social science theories of medicalization [43], clinicians explained that even informal “framing” language could unnecessarily “label” care users and divert from in-depth contextualized analysis.

Translating these clinical aspirations into everyday practice posed significant challenges. Clinicians noted that individuals living in precarious life circumstances were unlikely to seek help due to both access barriers and their estrangement from a medical system seen as dominated by the privileged. Those who did visit community clinics, often referred by schools, arrived having had negative experiences with counselling at school. As those leading psychiatric reform explained, during the dictatorship, cognitive-behavioral theories gained traction in schools more to maintain order than to help students [44]. Although changing, this trend has persisted.

From young people’s perspectives, teacher-initiated referrals to psychologists were, by and large, not seen as a form of care. The emphasis on "behavior" in schools was viewed as demeaning, unjust, and even discriminatory (Box 1).

**Table:** Box 1: Common views among marginalized youth regarding referrals for therapy at 15 years of age

Mauricio: There is a psychologist at school, but it’s just to say they have one, to make it [the school] look good.
Flávio: The teacher sent me to the psychologist because I’m from the *favela*… Gabriel: You should hear them, they come to you with this *frescura* (fuss, excess) about adolescent behavior…they are the ones that are crazy! (Gabriel’s emphasis) Jair: Whether they send us to the director’s office or the psychologist, it’s the same. They peg us, kids from the *favela.* Cintia: I am normal, but I would never talk to the counsellor even if I had a problem. They [school staff] tell each other everything, It’s just a ping-pong game of *fofocas* (gossip) there.

Youth noted the uneven distribution of blame-inducing behavioral terms like "inattention," "agitation," and "aggression,” and explained these were more likely to be used by psychologists and teachers with economically and racially minoritized youth than with wealthy and/or white youth (reference). Jair, who identified as black, recounted,

My Portuguese teacher came into the classroom. I don't know what the other [students] were doing, but I have a deep voice. Instead of reprimanding all the kids, she pegged me. She said all this stuff to me… she said she was disgusted by my face… could not stand me anymore. …I never did anything to her. And then I was sent to talk to the psychologist, who just asked me about my behaviors … and didn’t explain anything.

Like Jair, others asserted that the psychologist’s role should be explanatory rather than questioning, prescriptive, or subtly blaming. For some, disciplinary uses of school psychologists constituted a tipping point for withdrawing from school altogether.

### Challenging discrimination

While most students did not willingly return to the school psychologist, a unique minority did. What they sought was not merely care or symptom-reduction but justice for having been the target of discriminatory practices. They viewed the clinic as a “place for wealthy people,” and the therapist as a representative of institutional authority. Gabriel, for instance (Box 1), eventually returned to the school psychologist to make the explicit point that the problem for which he had been referred did not lie with him. Those who persisted*,* like him, sometimes received referrals to outpatient public sector providers, who tended to be psychodynamic in orientation and critical of behavioral reductionism, as shown in Box 2.

**Table:** Box 2: Critical views underpinning psychodynamic approaches

Eduardo, psychiatrist: “The rise in certain diagnoses like depression is not linked to ‘improved’ diagnostic systems of the DSM-IV and ICD-10, but is, rather, the result of giving the pharmaceutical industry free reign to [market] medications. [It would be over-simplified to say] … that attention deficit problems are related to [neurological problems only]. You wouldn’t have to leave … your clinic, take the time to go to the parents, the teachers, to visit the school and work with a context.”
Simone, psychologist, “Those in schools can be very quick to tell us, ‘This child has inattention.’ As if this were a ‘condition.’ But this is simplistic. The tensions between teachers and students are very intense.”
Bernardo, psychiatrist: *[Indisciplinados*, or students who lack discipline and are often told they have “inattention,” are] “suffering not from.. a psychopathology but from the scars caused by Brazil’s political history.”

The unstructured therapeutic methods providers typically used meant being open to young people’s confrontations. Diogo (Box 3), for example, used the clinic to “educate” the psychiatrist, a member of the elite (“they”), about the life experiences he had in common with other marginalized youth (“us”). Other young people recounted being “direct” and “angry” with the clinician. What needed changing, they added, was the school itself or even, as one young person said, “the way money corrupts people.” One psychiatrist said it was unrealistic to expect a clinical interaction infused with ease or understanding; the absence of conflict, he added, likely indicated that only surface-level therapy was taking place.

Resisting behavioral language was an important component of clinical confrontations. Diogo, for example, reframed his “temper” as a legitimate response to injustice, and Jair explained that true understandings of his “storm-like emotions” required “saying it like it is” (Box 3). Reframing often entailed analyses that situated mental experiences in a life narrative and social context. Figuring prominently in this reframing was not poverty-induced suffering, a core focus of the social determinants of health literature, but *preconceitos* (prejudices, discrimination), conflict, and disempowerment. For Marisa, encountering a therapist who could willingly accept the limits of their own understanding was key (Box 3).

**Table:** Box 3: Therapy as a place to practice conflict and challenge discrimination

Diogo: I told him [psychiatrist], you know, I explained to him… I know I can lose my temper… But what really makes me explode is when I am unjustly judged, like what happened at work when I was [unjustly] accused of stealing. They make assumptions [about us]. (Diogo’s emphasis)
Jair: You know that saying…’when the person who is too calm explodes, it’s a storm.’ That’s me. That’s why I like to talk things through. It’s not possible to have an open dialogue with just anyone, but still… it’s good to try to discuss…. even with the psychologist, and sometimes that means saying it how it is. (Jair’s emphasis) Marisa: I was surprised that the psychologist asked me to explain things, to teach her; she spoke to me like an equal. But are lives are so different, and she understands that now.

Only a few young people debated discrimination explicitly in the clinic. Yet among those who challenged behavioral framings and were referred for mental health care, clinic-attenders spoke about discrimination in everyday life more intensely than non-clinic-attenders.

Building on these insights, the epidemiological analysis assessed the relationship between use of therapy and discussions of discrimination. Seeing a therapist in 2001 and any time up to 2001 were positively associated with reporting discrimination in both 2004–05 and 2012–13. Use of therapy in 2012–13 was associated with reporting discrimination in 2012–13 (Table 3). Regression analysis shows that these relationships remained significant after controlling for confounders, with RRs ranging from 1.3 to 2.3 (Table 4). The epidemiological analysis showed that young people who articulated their problems in narrative terms reported experiences with discrimination in 2004–05 more frequently than those who understood their problems behaviorally and those who did not go to therapy (Table 3). After controlling for confounders, young people who understood their problems in narrative terms were 2.5 times more likely to report discrimination than those who did not go to therapy (Table 4). This is noteworthy since low income and non-white youth were more likely to frame their problems in behavioral terms (data not shown), most likely because this is how they were managed in schools. Framing language was not associated with reporting discrimination in 2012–13.

**Table 3:** Patterns of therapeutic use and reported discrimination and political activity

	Reporting experiences with discrimination in the last year				Reported political activity													
Survey	2004–05		2012–13		2001		2004–05										2006	
Variable	One or more forms of discrimination		One or more forms of discrimination		Political protest, petition, and/or voting		Political protest or petition		Political groups in school, university or political party		Neighborhood association		Political campaign		Youth groups		Political protest or petition	
	*n*	%	*n*	%	*n*	%	*n*	%	*n*	%	*n*	%	*n*	%	*n*	%	*n*	%
Use of therapy in last year (2001)	0.001		< 0.001		< 0.001		< 0.0001		< 0.0001		< 0.001		< 0.001		0.01		< 0.001	
No	120	11	149	17	453	31	325	31	134	13	28	3	171	16	209	29	406	46
Yes	21	16	24	34	61	54	43	54	24	30	7	9	22	28	34	43	46	68
Lifetime use of therapy (2001)	0.008		< 0.001		0.008		< 0.001		0.004		0.10		0.09		0.05		0.04	
No	71	9	99	15	271	31	233	29	96	12	21	3	126	16	228	28	303	45
Yes	64	19	74	26	142	39	135	37	62	18	15	4	67	20	115	34	150	53
Use of therapy in last year (2004–05)	0.09		0.02				0.007		0.016		0.7		0.04		0.3		0.03	
No	122	11	145	17			335	31	142	13	33	3	178	17	319	30	391	47
Yes	14	18	19	28			36	46	18	23	3	4	20	25	28	35	40	61
Use of therapy in last year (2012–13)			< 0.001															
No			137	16														
Yes			41	35														
Framing used in explaining need for therapy at any point up to 19 years (2001)	< 0.0001		< 0.001		0.09		< 0.001		0.005		0.5		0.1		0.03		0.04	
No therapy	71	9	99	15	539	61	233	29	96	12	21	3	126	16	228	28	303	45
Behavioral	22	16	32	29	95	66	41	30	19	14	4	3	20	15	39	29	51	48
Narrative	35	23	32	22	126	69	76	47	35	22	7	4	36	22	63	39	81	57

**Table 4:** Regression analysis for therapeutic use and discrimination and politicization

	Relative risk (RR) (CI 95%)															
	Reporting experiences with discrimination in the last year				Reported political activity											
Survey	2004–05		2012–13		2001		2004–05								2006	
Variable	One or more forms of discrimination		One or more forms of discrimination		Participation in either political protest or voting		Political protest or petition		Political groups in school, university, or political party		Neighborhood association		Political campaign		Political protest or petition	
	Crude	Adjusted^a^	Crude	Adjusted^a^	Crude	Adjusted^b^	Crude	Adjusted^a^	Crude	Adjusted^a^	Crude	Adjusted^a^	Crude	Adjusted^a^	Crude	Adjusted^a^
Use of therapy in last year (2001)	0.17	0.04	< 0.001	< 0.001	< 0.001	0.5	< 0.001	< 0.001	< 0.001	< 0.001	< 0.0001	< 0.001	< 0.001	0.01	< 0.01	0.2
No	Ref.	Ref.	Ref.	Ref.	Ref.	Ref.	Ref.	Ref.	Ref.	Ref.	Ref.	Ref.	Ref.	Ref.	Ref.	Ref.
Yes	1.4 (0.9–2.4)	1.8 (1.0 -3.0)	2.0 (1.4–2.9)	2.3 (1.6–3.3)	1.3 (1.1–1.4)	1.1 (1.0–1.3)	1.8 (1.4–2.2)	1.5 (1.2–1.9)	2.4 (1.6–3.4)	2.2 (1.5–3.3)	3.3 (1.5–7.4)	4.2 (2.0–8.8)	2.2 (1.4–3.5)	1.9 (1.2–3.0)	1.5 (1.1–2.0)	1.3 (0.9–1.7)
Lifetime use of therapy (2001)	< 0.001	< 0.001	< 0.001	0.001	0.2	0.3	< 0.01	0.07	0.008	0.04	0.03	0.07	0.1	0.2	0.3	0.2
No	Ref.	Ref.	Ref.	Ref.	Ref.	Ref.	Ref.	Ref.	Ref.	Ref.	Ref.	Ref.	Ref.	Ref.	Ref.	Ref.
Yes	2.1 (1.5–3.0)	2.3 (1.6–3.2)	1.7 (1.3–2.3)	1.7 (1.2–2.4)	1.1 (1.0–1.2)	1.0 (1.0–1.1)	1.4 (1.2–1.71	1.2 (1.1–1.5)	1.5 (1.1–2.1)	1.4 (1.0–2.0)	1.7 (.9–3.3)	1.8 (1.0–3.4)	1.3 (0.9–1.7)	1.3 (0.9–1.7)	1.2 (1.0–1.5)	1.1 (1.0–1.4)
Use of therapy in last year (2004–05)	0.08	0.02	0.02	0.004			0.003	0.04	0.01	0.04	0.7	0.5	0.04	0.03	0.02	0.2
No	Ref.	Ref.	Ref.	Ref.			Ref.	Ref.	Ref.	Ref.	Ref.	Ref.	Ref.	Ref.	Ref.	Ref.
Yes	1.6 (1.0–2.6)	1.8 (1.1–2.0)	1.7 (1.1–2.5)	1.8 (1.2–2.7)			1.5 (1.1–1.9)	1.3 (1.0–1.7)	1.7 (1.1–2.7)	1.6 (1.0–2.5)	1.2 (0.4–4.0)	1.4 (0.5–4.4)	1.5 1.0–2.3)	1.6 (1.1–2.3)	1.3 (1.0–1.6)	1.2 (0.9– 1.4)
Use of therapy in last year (2012–13)			< 0.001	< 0.001												
No			Ref.	Ref.												
Yes			2.2 (1.6–2.9)	2.2 (1.6–2.9)												
Framing used in explaining need for therapy at any point up to 19 yrs (2001)																
No therapy	Ref.	Ref.	Ref.	Ref.	Ref.	Ref.	Ref.	Ref.	Ref.	Ref.	Ref.	Ref.	Ref.	Ref.	Ref.	Ref.
Behavioral	1.8 (1.1–3.0) 0.01	2.0 (1.2–3.2) 0.007	2.0 (1.3–2.9) < 0.001	1.8 (1.2–2.7) < 0.01	1.1 (0.9–1.4) 0.5	1.0 (0.8–1.3) 0.9	1.0 (0.8–1.5) 0.9	1.0 (0.7–1.4) 0.8	1.2 (0.7–1.9) 0.5	1.0 (0.6–1.7) 0.9	1.1 (0.3–3.8) 0.8	1.2 (0.4–3.9) 0.8	0.9 (0.6–1.5) 0.8	0.9 (0.6–1.5) 0.7	1.1 (0.8–1.4) 0.7	0.9 (0.7–1.3) 0.9
Narrative	2.5 (1.6–3.7) < 0.001	2.5 (1.6–3.8) < 0.001	1.5 (1.0–2.3) 0.04	1.6 (1.0–2.5) 0.03	1.1 (0.9–1.4) 0.2	1.1 (0.9–1.4) 0.6	1.6 (1.2–2.1) < 0.001	1.4 (1.1–1.9) 0.01	1.8 (1.2–2.7) < 0.001	1.7 (1.1–2.6) < 0.01	1.6 (0.7–3.3) 0.4	1.6 (0.6–3.4) 0.4	1.4 (1.0–2.0) 0.06	1.5 (1.0–2.2) 0.04	1.3 (1.0–1.6) 0.07	1.2 (0.9–1.5) 0.2

^a^Adjusted for family income at birth, maternal education at birth, household asset index, participant’s racial identification, family income at 2004–05 follow-up

^b^Adjusted for family income at birth, maternal education at birth, household asset index, participant’s racial identification, family income at 2001 follow-up 2

### Engaging politics

As young people practiced confrontation, debate, and assertion in the clinic, so too did they intensify broader forms of social and political engagement, both informally and in youth groups, neighborhood associations, and school-based activities. As they widened their social activities, they also tended to remain in school, beyond the time when most of their peers of similar socio-economic backgrounds had dropped out. The impetus to become more socially active was turbulent and not merely about seeking support. On the contrary, facing conflict and discrimination were vital. For example, Mauricio and Flávio described how important it became to grapple with their fear of being targeted by shopkeepers when going to the city center, a common hangout for middle-class teens.

Over the years, young people’s practices and experiences cycled in micro-iterations, from widened social engagement to increased exposure to conflict to more emotional distress, a return to the clinic, and more desire for social participation in turn. Throughout these cycles, they explored their emotions more freely, delving into relational dynamics that fueled their impetus to challenge norms, while continuing to ask the therapist to provide “inside knowledge” on the innerworkings of structures of power. They began to consider not just how social life shaped them, but also how they might intervene in the world. Interest in social activism broadened from the “micropolitics” of everyday life to more formal activities from which marginalized and minoritized youth often felt alienated, such as protests, petitions, student government, political parties, and voting. Thus, the social realm that came to matter most centered on political mechanisms of change.

Investigating this finding in the larger sample, the epidemiological analysis found that use of therapy in 2001 was associated with all 7 indicators of social and political activity measured in the 2001, 2004–05, and 2006 surveys (Table 3). In the regression analysis, 4 indicators of political activity remained associated with use of therapy in 2001 with RRs of 1.5, 2.2, 4.2, and 1.9, respectively (Table 4). In the unadjusted analysis, lifetime use of therapy up until 2001 was associated with 5 indicators of political activity, and in the adjusted analysis, one of these remained associated: participation in school. Use of therapy in 2004–05 was associated in the unadjusted analysis with 4 indicators of political activity and with 3 indicators in the adjusted analysis. Using a narrative framing to understand mental distress was associated with four indicators of political activity in the unadjusted analysis and two indicators remained significant in the adjusted analyses (Tables 3, 4).

Our ethnographic observations indicated that the social and political effects of therapy were more salient and meaningful for marginalized and minoritized youth. To explore this result quantitatively, we used one outcome variable, political activity in the context of the school, university, or political party in 2004–05, because marginalized youth spoke most intensely about discrimination in schools and because this variable remained significant in the regression and had a larger sample size. Results shown in Table 5 suggest that seeing a therapist in 2001 was more strongly associated with political activity in 2004–05 for young people living in situations of increased precarity. Youth who visited a therapist in 2001 who had mothers with less than 9 years of education at birth were 3.3 times more likely to engage in political activity than those who had not seen a therapist, whereas the RR among those with mothers who had 8 or more years of education was 1.4. Youth who visited a therapist in 2001 whose families had an asset index in 1984 of less than 3.56 were 4.1 times more likely to engage in political activity than those who had not seen a therapist, whereas the RR among those whose families had an asset index of 3.55 or above was 1.7. No effect modification was found for young person’s self-identified race.

**Table 5:** Effect modification of relationship between use of therapy (2001) and political activity in school, university, or political party (2004–05)

		Rate ratios (RR) (CI 95%)		*p* value for interaction
		Political participation in school, university, or political party (2004–05)		
		Maternal schooling at birth (1982)		
Use of therapy in the last year (2001)		< 9 years	> 8 years	
No	Ref.	Ref.	Ref.	
Yes	2.4 (1.6–3.4) < 0.001	3.3 (2.0–5.3) < 0.001	1.4 (0.8–2.5) 0.2	0.03
		Asset index in 1984		
Use of therapy in the last year (2001)		< 3.56	> 3.55	
No	Ref.	Ref.	Ref.	
Yes	2.4 (1.6–3.4) < 0.001	4.1 (2.4–7.1) < 0.001	1.7 (1.1–2.8) 0.03	0.02
		Participant’s self-identified race		
Use of therapy in the last year (2001)		Black, brown, other	White	
No	Ref.	Ref.	Ref.	
Yes	2.4 (1.6–3.4) < 0.001	3.3 (1.4–7.7) 0.005	2.2 (1.5–3.4) < 0.001	0.40

In the ethnographic follow-up initiated in 2020 with 20 participants from the original sample, a bit more than half reported using therapy at some point in their life, and about 60% had continued using therapy on and off throughout their adult years. Comparative analysis of those who had continued using therapy in adulthood with those who did not suggest that the socio-political potential of therapy persisted in adulthood. Unlike those who relied mostly on medication or family support for their mental health, participants who engaged in socially meaningful therapy became community leaders, activists, or aspiring politicians. Importantly, they also viewed their emotional struggles as integral to their political and social commitments.

## Discussion

Though the socially attuned therapies described in this paper were not common or mainstream, they hold considerable potential. Young people who returned to the clinic, despite intense feelings of disappointment and mistrust, used the therapeutic encounter to engage in social and political debate and explore new modes of agency. Clinical interactions came to center not on treatment of disorder or symptom-reduction, but on crafting self-worth, political awareness, and social influence [45]. What seems to have made a difference for young people were therapists who responded flexibly, recognized the limits of their own positionality, and maintained dialogic openness to unstructured reflection and the productivity of confrontation.

Because social and political activity often increased emotional struggle [46, 47], distress often became constitutive of the therapeutic process itself [48]. Also key were young people’s repudiation of behavioral language and their ongoing reframing of mental distress as inextricably social, relational, and political [49]. The relationship between therapy and both socio-political participation and reporting discrimination remained statistically significant for most outcome variables after controlling for confounders, suggesting that therapy had an independent effect on socio-political practice. Effect modification showed that the relationship between use of mental health services and socio-political engagement was stronger for those with less formal education and family income but not for those who identified as black or brown. This points to the deeply structured nature of racialization and racism.

The study has several strengths. The ethnographic material generated new and innovative hypotheses and provided interpretive depth. The use of population-based sampling for both the ethnographic and epidemiological samples, and the exploration of social life outside the clinic, brought elements to the table not usually present in clinic-based studies. The prospective design provided insights into cause-and-effect relationships and change over time. Multiple quantitative measures of therapeutic use and political activity at different time-points show a consistency of trends, and strong measures of co-variates allowed for effective controlling of confounders.

Several limitations should be noted. Since the clinical encounter was not observed but rather relayed by young people and therapists, clinical characteristics could not be directly confirmed. Loss to follow-up, while not significant for a longitudinal study of this duration, likely led to an under-representation of low income and racially marginalized individuals. With regards to causality, young people who experienced socially productive therapy were likely self-selected, as many had been involved in socio-political activities before therapy. This does not mean, however, that the clinic did not have influence independent of young people’s contributions, as noted. The ethnographic data suggest that the relationship between therapy and sociopolitical activity was cyclical and synergistic. Thus, “self-selection” should in this sense be considered a therapeutic opportunity.

While not mainstream, the relationship between therapy and social and political engagement has a long and varied history [50, 51]. Scholars in social work, political psychology, feminist therapeutics, social psychiatry, medical anthropology, and critical global health have repeatedly called for approaches that tackle the root social causes of ill health [52, 53]. Yet most initiatives addressing the social determinants of health focus on supporting marginalized individuals and families to access care and adopt “healthier behaviors” [54]. Even when the health sector collaborates with the social welfare, housing, employment, or education sectors, it is usually to improve access to services rather than to push for policies that structurally eliminate root causes [55]. The recent rise in “social prescribing” practices in mental health care has tended to transform “the social” into a matter of individual responsibility, setting a path for the state and health services to be relieved of their responsibilities [56]. In the absence of sustained structural transformation, such initiatives remain limited and potentially harmful [57].

Results from this study resonate with calls made by LASM scholars for a shift from static and individualized understandings of the social to ones that are dynamic and dialectical [58–60]. Such a shift prioritizes a view of the social realm as not only “risk-laden” but as a source of inspiration, shifting from a politics of “denouncement”—that is, “diagnosing” social problems—to one inspired by the ethical commitment to build a more just society [61]. A dynamic view of the social also pinpoints possibilities for change by elucidating questions of agency. Structural transformations can ensue not only through macro-level policies but also from grassroots experiments in democratic governance, epistemic justice, critical consciousness, co-production initiatives, and in everyday modes of living and relating to others [62–65]. As this paper has shown, frank discussions of the emotional damages of the social and political realm played an important role in motivating young people’s desire to practice change-making and hold their therapists accountable to doing the same [66].

## Data Availability

The questionnaires and interviewer guides from all follow-up visits are available in electronic formats at [http://www.epidemio-ufpel.org.br/site/content/coorte_1982/questionarios.php]. Applications to use the data should be made by contacting the researchers of the 1982 cohort and completing the application form for the Pelotas Birth Cohorts available at [http://www.epidemio-ufpel.org.br/site/content/estudos/formularios.php].
